# Levels of Tetrodotoxins in Spawning Pufferfish, *Takifugu alboplumbeus*

**DOI:** 10.3390/md21040207

**Published:** 2023-03-25

**Authors:** Masaki Asano, Chihiro Ishizaki, Taiga Tomonou, Masato Kihara, Masaaki Ito, Shino Yasukawa, Kyoko Shirai, Hikaru Oyama, Shin Izawa, Reona Kawamura, Kanae Saito, Rei Suo, Ryota Nakahigashi, Masaatsu Adachi, Toshio Nishikawa, Haruo Sugita, Shiro Itoi

**Affiliations:** 1 Department of Marine Science and Resources, Nihon University, Fujisawa 252-0880, Japan; 2 Laboratory of Organic Chemistry, Graduate School of Bioagricultural Sciences, Nagoya University, Chikusa, Nagoya 464-8601, Japan; 3 Graduate School of Pharmaceutical Sciences, Tohoku University, Aoba, Aramaki, Aoba-ku, Sendai 980-8578, Japan

**Keywords:** moray eel, pufferfish, sex difference, 5,6,11-trideoxyTTX, TTX, TTX tolerance

## Abstract

Tetrodotoxin (TTX), also known as pufferfish toxin, is an extremely potent neurotoxin thought to be used as a biological defense compound in organisms bearing it. Although TTX was thought to function as a chemical agent for defense and anti-predation and an attractant for TTX-bearing animals including pufferfish, it has recently been demonstrated that pufferfish were also attracted to 5,6,11-trideoxyTTX, a related compound, rather than TTX alone. In this study, we attempted to estimate the roles of TTXs (TTX and 5,6,11-trideoxyTTX) in the pufferfish, *Takifugu alboplumbeus*, through examining the location of TTXs in various tissues of spawning pufferfish from Enoshima and Kamogawa, Japan. TTXs levels in the Kamogawa population were higher than those in the Enoshima population, and there was no significant difference in the amount of TTXs between the sexes in either population. Individual differences were greater in females than in males. However, the location of both substances in tissues differed significantly between sexes: male pufferfish accumulated most of their TTX in the skin and liver and most of their 5,6,11-trideoxyTTX in the skin, whereas females accumulated most of their TTX and 5,6,11-trideoxyTTX in the ovaries and skin.

## 1. Introduction

Tetrodotoxin (TTX), a highly potent neurotoxin, was thought to be specific to pufferfish, but since its complex chemical structure was revealed [1,2,3], the substance has been detected in many marine organisms, such as the toxic goby *Yongeichthys criniger* [4], xanthid crabs [5], blue-ringed octopus [6], and flatworms [7,8,9,10,11], as well as amphibians [12,13] and terrestrial flatworms [14]. Since then, many TTX analogues including deoxyTTXs, dideoxyTTXs, and 5,6,11-trideoxyTTX have also been discovered in these TTX-bearing organisms [13,15,16,17,18,19,20,21].

It has been suggested that pufferfish are unable to synthesize TTX themselves and accumulate high concentrations of TTX through bioaccumulation by preying on TTX-bearing organisms lower in the food web [22]. The biological functions of TTXs in pufferfish remain unclear, although it has been suggested that TTX is utilized as a biological defense against predators [23] and as a pheromone-like substance [24,25,26]. Recently, it has been shown that pufferfish larvae of the genus *Takifugu* efficiently protect themselves from predators via the localization of maternal TTX on their own body surface [27,28]. As pufferfish grow, TTX is accumulated in the liver, ovaries, and other internal organs, but in many pufferfish species, TTX is still distributed in the skin, where it is thought to offer predator protection [22].

**Figure 1:**
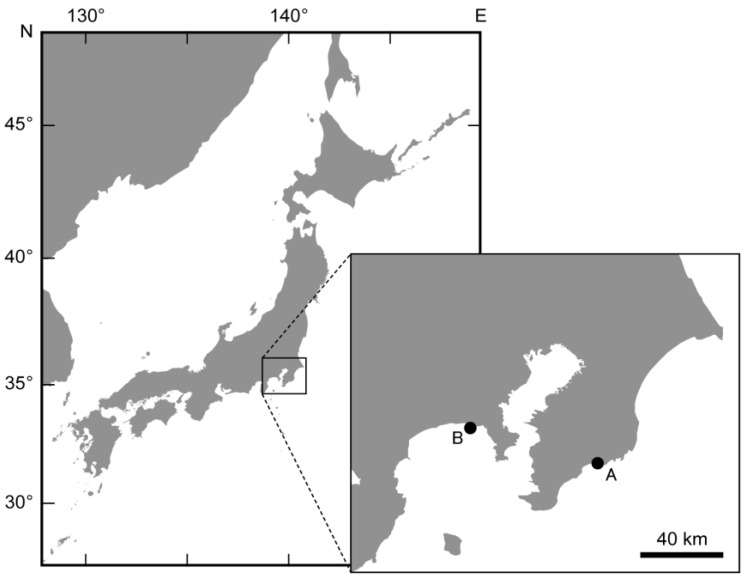
Sampling localities for the pufferfish *Takifugu alboplumbeus* used in this study. Sampling details can be seen in the inset: A, Kamogawa, Chiba Prefecture; B, Enoshima, Kanagawa Prefecture, Japan.

On the other hand, although pufferfish possess TTX, it seems unlikely that they would have no potential predators. However, moray eels *Gymnothorax kidako* have recently been observed preying on spawning pufferfish, *Takifugu alboplumbeus* (previously *Takifugu niphobles*). We have confirmed that moray eels prey on the pufferfish *T. alboplumbeus* during spawning at various locations such as Enoshima Island in Kanagawa Prefecture and Kamogawa in Chiba Prefecture, Japan (Figure 1). In these areas, the pufferfish have been known to crowd into spawning grounds by the thousands or tens of thousands to spawn near high tide during new and full moons of May and July [29,30]. There have been no reports of moray eels possessing TTX or being resistant to it; however, moray eels have been observed preying on the pufferfish *T. alboplumbeus* in various locations. In general, it cannot be ruled out that morays may have died several hours after predation without TTX tolerance. Indeed, it has been reported that a blue-lined octopus *Hapalochlaena fasciata* was found in the esophagus of dead adult green sea turtles with no apparent external damage, and the sea turtles were thought to have died due to TTX of the octopus [31]. Thus, the active predation of the moray eels on the pufferfish aroused interest in whether the moray eels have a TTX tolerance or whether the amount of TTX in the spawning pufferfish has changed.

In this study, we investigated the amount of TTX and 5,6,11-trideoxyTTX, and the distribution of these substances in various tissues of the spawning pufferfish *T. alboplumbeus*.

## 2. Results

### 2.1. Sex Ratio and GSI of the Spawning Pufferfish

The sex ratio of spawning pufferfish at Enoshima (*n* = 360) and Kamogawa (*n* = 90), Japan (Figure 1) in June 2022 was 9:1 male to female at both sites. Of the individuals collected, 11 males and 9 females from Kamogawa and 14 males and 12 females from Enoshima were transported to the laboratory and gonadosomatic index (GSI), calculated as gonad weight as a proportion of the total body weight, was calculated. The GSI of females at Kamogawa ranged from 5 to 22 and males from 10 to 20, while females at Enoshima ranged from 5 to 29 and males from 12 to 20 (Figure S1, Supplementary Material).

### 2.2. Amount of TTX and 5,6,11-TrideoxyTTX in the Pufferfish

**Figure 2:**
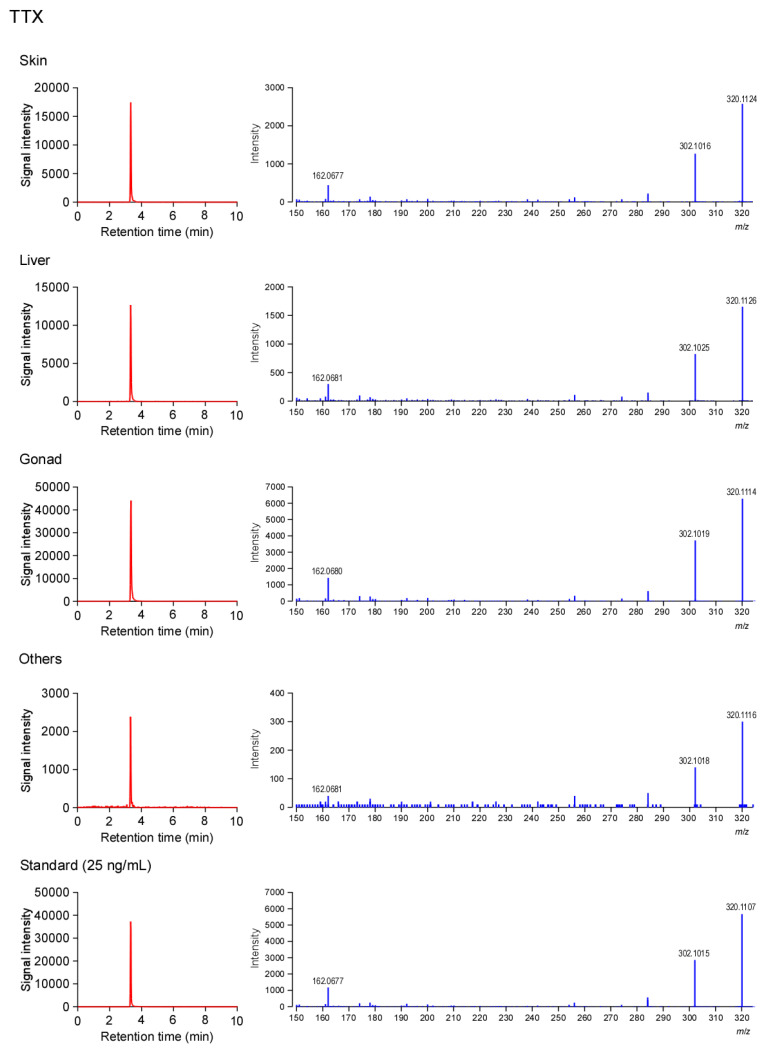

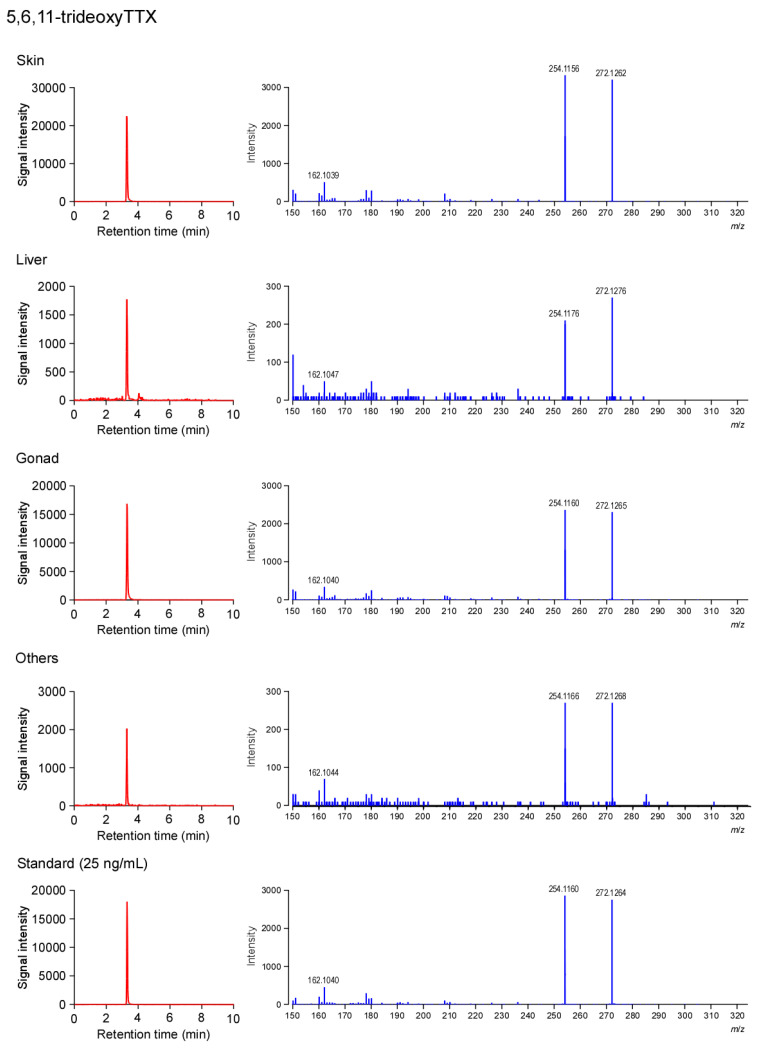
Sampling LC-MS/MS patterns of the TTX and 5,6,11-trideoxyTTX from the pufferfish *Takifugu alboplumbeus*, and 25 ng/mL standard. Left panels represent LC-MS/MS chromatograms, while the right panels represent the precursor and product ion mass spectra for TTX.

LC-MS/MS analysis demonstrated that TTX and 5,6,11-trideoxyTTX compounds were detected in all the individuals collected from Kamogawa and Enoshima (Figure 2).

**Figure 3:**
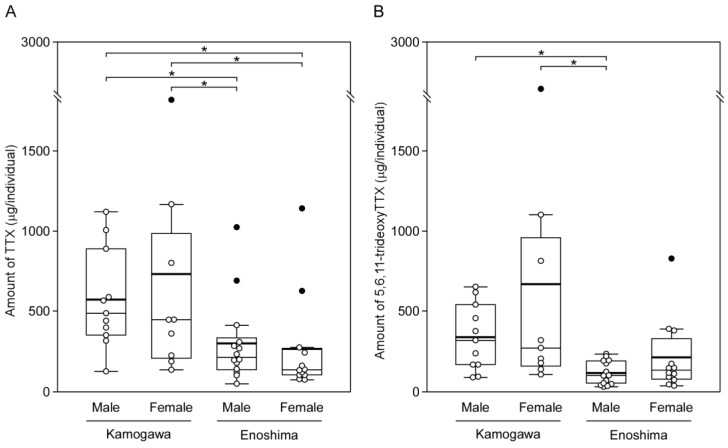
Box and whisker plot comparing the mean and median TTX and 5,6,11-trideoxyTTX amounts (μg/individual) for the pufferfish *Takifugu alboplumbeus* sampled from two localities in the Japanese Islands. (**A**), TTX; (**B**), 5,6,11-trideoxyTTX. The upper and lower edges of each box represent the value of the 3rd and 1st quartile, respectively, while the length of the whiskers reflects the variability outside these two quartiles. The thin and bold line within the boxes represent the values of median (2nd quartile) and mean, respectively. Circles represent the measured values, of these, black circles represent outliers. Asterisks indicate significant difference at *p* < 0.05.

As shown in Figure 3, in the Kamogawa population, TTX levels in males were 573 ± 294 µg/individual and levels in females were 734 ± 799 µg/individual, with no significant difference between the two (Steel–Dwass test, *p* > 0.05). Similarly, in the Enoshima population, TTX levels for males and females were 301 ± 252 and 267 ± 301 µg/individual, respectively, without significant differences between the two groups (*p* > 0.05).

Similar patterns were observed in 5,6,11-trideoxyTTX: in the Kamogawa population, the amount of 5,6,11-trideoxyTTX in males was 337 ± 197µg/individual and in females was 667 ± 846 µg/individual, and no significant difference was found between the sexes (*p* > 0.05). In the Enoshima population, 5,6,11-trideoxyTTX levels for males and females were 117 ± 69 and 211 ± 216 µg/individual, respectively, not significantly different between the two (*p* > 0.05).

However, TTX levels were significantly higher in Kamogawa males and females than in their Enoshima counterparts (*p* < 0.05). 5,6,11-trideoxyTTX levels also tended to be higher in the Kamogawa population than in the Enoshima population: significant differences were observed between males in the Enoshima population and both sexes in the Kamogawa population (*p* < 0.05).

### 2.3. Tissue Distribution of TTX and 5,6,11-TrideoxyTTX

Tissue localization of TTX differed significantly between males and females (*p* < 0.05), and this pattern was consistent in both the Kamogawa and Enoshima populations (Table 1, Figure S1). In the Kamogawa population, males localized 36.8 ± 21.2% and 31.2 ± 13.8% of TTX in the skin and liver, respectively, whereas females localized 54.3 ± 15.0% of TTX in the ovaries and 25.0 ± 11.1% in the skin. Similarly, in the Enoshima population, males localized 44.4 ± 18.4% and 33.7 ± 12.6% of TTX in the skin and liver, respectively, whereas females localized 59.2 ± 18.3% in the ovaries and 30.8 ± 13.5% in the skin.

Tissue localization of 5,6,11-trideoxyTTX also differed significantly between males and females (*p* < 0.05), but no geographical differences were observed (Table 1, Figure S1). In the Kamogawa population, males localized 69.9 ± 18.8% of 5,6,11-trideoxyTTX in the skin, whereas females localized 46.7 ± 20.8% and 47.2 ± 18.2% of 5,6,11-trideoxyTTX in the ovaries and skin, respectively. Similarly, in the Enoshima population, males localized 71.4 ± 17.0% of 5,6,11-trideoxyTTX in the skin, whereas females localized 54.7 ± 23.8% and 38.8 ± 20.4% of 5,6,11-trideoxyTTX in the ovaries and skin, respectively.

**Figure 4:**
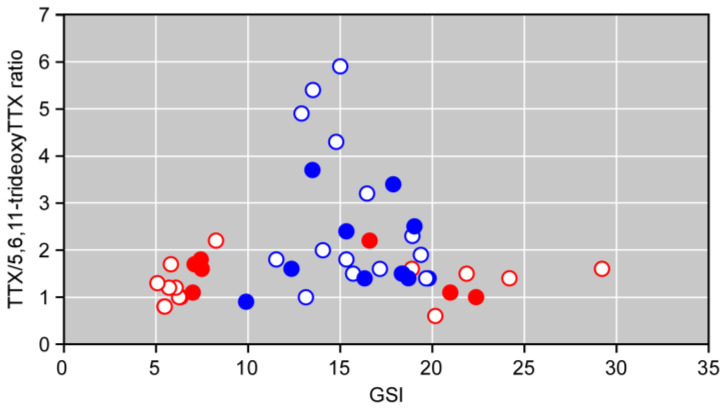
Distribution of GSI and TTX/5,6,11-trideoxyTTX ratios of spawning male and female pufferfish *Takifugu alboplumbeus* at Kamogawa and Enoshima. Blue and red circles represent the values for males and females, respectively, whereas open and closed circles represent the values for individuals in Enoshima and Kamogawa, respectively.

The ratio of TTX to 5,6,11-trideoxyTTX did not differ between the Kamogawa and Enoshima populations (*p* > 0.05), with more TTX than 5,6,11-trideoxyTTX in all tissues except skin (Table 2). In the whole body, the ratios were 2.0 ± 0.8 and 1.4 ± 0.5 for males and females in the Kamogawa population (*p* > 0.05), respectively, whereas those for males and females were 2.8 ± 1.6 and 1.3 ± 0.4 in the Enoshima population (*p* < 0.05), respectively. At both Enoshima and Kamogawa sites, females had a TTX/5,6,11-trideoxyTTX ratio of 1–2 for both GSI below 10 and above 15, whereas males had a GSI clumped between 10 and 20, and many of them showed higher TTX/5,6,11-trideoxyTTX ratios compared to females (Figure 4).

## 3. Discussion

It has been reported that a species of the *Takifugu* pufferfish exhibit a pattern of seasonal variation in TTX similar to that of the pufferfish *T. alboplumbeus* [26]: *Takifugu poecilonotus* (currently *Takifugu flavipterus*) localize TTX to the skin and ovaries in females and to the skin and liver in males prior to the spawning period [32]. Both *T. alboplumbeus* and *T. flavipterus* have been known to localize large amounts of TTX to the skin [22], suggesting a common role for TTX in pufferfish of the genus *Takifugu* in localizing TTX to the skin. Although *Takifugu* larvae have been known to protect themselves by localizing maternal TTX on the body surface [27,28], some species of the genus *Takifugu*, such as *Takifugu rubripes*, have been known to lose TTX localization in the skin as they grow [22], and the process of variation in the localization mechanism of TTXs among species of the genus *Takifugu* is interesting from an evolutionary aspect.

Although individuals with large amounts of TTX were detected, the amount of TTX in both sexes of the pufferfish *T. alboplumbeus* participating in spawning in Kamogawa and Enoshima was lower compared to the pufferfish caught in non-spawning grounds during the spawning period [26]. In both spawning grounds, females with GSI below 10 and above 15 were observed: females with GSI below 10 were post-spawning individuals, whereas those above 15 were likely pre-spawning or actively spawning. Although TTX levels decrease in association with spawning, it appears that individuals with extremely large amounts of TTX continue to possess large amounts even after spawning. In contrast, males had lower amounts of TTX localized in the skin compared to previously reported data [26], suggesting that TTX may have been released from the skin at/around the spawning grounds. This species of pufferfish is known to have TTX glands on their body surface [33], from which they may have released large amounts of TTX.

On the other hand, male pufferfish caught on the spawning grounds localized nearly 70% of total 5,6,11-trideoxyTTX throughout their bodies on their skin. Recently, it has been reported that the pufferfish *T. alboplumbeus* and the green spotted pufferfish *Dichotomyctere nigroviridis* responded to and were attracted to 5,6,11-trideoxyTTX, whereas they did not respond to TTX, based on electrophysiological and behavioral experiments, and that their olfactory organs were involved in this response [34,35]. There is speculation that in *T. alboplumbeus*, TTX has a pheromone-like effect, based on the significant decrease in TTX after the spawning period [26] and the known attraction effect with TTX in both *T. alboplumbeus* [24] and *T. rubripes* [25]; but this was probably an observation of the response to 5,6,11-trideoxyTTX, which is present simultaneously with TTX. 5,6,11-trideoxyTTX is extremely low in toxicity [36], and it is unlikely that pufferfish possess it to protect themselves from predators. Nevertheless, the localization of a large amount of 5,6,11-trideoxyTTX on the body surface suggests that it has some role. Some pufferfish, such as *T. alboplumbeus*, *T. flavipterus* and *Takifugu vermiculare* (previously *Takifugu vermiculare radiatum*), are known to release TTX from TTX glands on their body surface when stimulated [23,33], and it is assumed that they simultaneously release 5,6,11-trideoxyTTX. Therefore, it seems reasonable to assume that *T. alboplumbeus* responds 5,6,11-trideoxyTTX as an aggregation pheromone-like substance during spawning. However, the mechanism of release, including the selectivity of TTX and 5,6,11-trideoxyTTX release, remains unknown.

The 5,6,11-trideoxyTTX release may be related to changes in the TTX/TDT ratio. The distribution pattern of the TTX/5,6,11-trideoxyTTX ratio and GSI differed between the males and females of the spawning *T. alboplumbeus*, suggesting a relationship with the spawning ecology. *Takifugu alboplumbeus* are known to spawn for approximately two months from late May along the southern coast of the Kanto region, Japanese Islands [29,30,37]. During the spawning period, there are 4–5 high/mid tides around the new/full moon when the pufferfish spawn, and spawning behavior is observed for about 5 days in each tide, or 20–25 days in a year [29,30,37]. The male-to-female ratio of spawning *T. alboplumbeus* is about 1:10–40, with an overwhelmingly high proportion of males [30,37], as in the case of this study. It is believed that females release all their eggs in a single spawning, whereas males release sperm many times during the spawning period [37], which was supported by the results of this study. Females release eggs containing a large amount of TTX and 5,6,11-trideoxyTTX when they spawn, so the TTX/5,6,11-trideoxyTTX ratio in their bodies did not change and GSI decreased, whereas males repeatedly release 5,6,11-trideoxyTTX through their skin to aggregate at spawning grounds, so individual differences in the TTX/5,6,11-trideoxyTTX ratio may have been greater. The repeated aggregation of male pufferfish to spawning grounds may not be for the sole purpose of sperm release, but to release 5,6,11-trideoxyTTX from the skin so that the population can effectively aggregate to the spawning grounds.

Thus, it is possible that the amount of TTX may decrease in spawning pufferfish. The behavior of moray eels preying on spawning pufferfish, which prompted this study, may be related to the targeting of these less toxic pufferfish. In our preliminary study, moray eels could not survive an intraperitoneal injection of 800 µg of TTX per kilogram of body weight (BW), but could survive 400 µg/kg of TTX (Preliminary study, Table S1), so it is possible that moray eels weighing 1 to 2 kg could survive a dose of 400 to 800 µg/kg of TTX. This TTX tolerance is about 80 times higher than that of non-toxic fish, such as the largescale blackfish *Girella punctata* [38], and comparable to that of non-toxic species of pufferfish, such as the brownback toadfish *Lagocephalus spadiceus* [38]. Based on the amount of TTX in the spawning pufferfish identified in this study, morays (1–2 kg BW) may be able to safely prey on about one or two individuals of pufferfish at Enoshima and one individual at Kamogawa. TTX content in pufferfish varies widely among individuals, and several pufferfish were found to contain more than 1 mg of TTX, so there is no doubt that it is dangerous for morays to prey on them. Further research is required on the mechanism of TTX tolerance in moray eels.

In conclusion, the localization of TTX and 5,6,11-trideoxyTTX in spawning individuals of the pufferfish *T. alboplumbeus* differs drastically between the sexes, and this difference may reflect their different roles in spawning ecology. Female pufferfish use TTX accumulated in their ovaries to protect their larvae, whereas male pufferfish may use 5,6,11-trideoxyTTX as a pheromone-like substance for efficient aggregation of the population to their spawning grounds. This study suggests that the observation of localization changes of TTX and its analogues related to life history, maturation stages, and ecological events in pufferfish may help us to better understand the biological roles of TTXs in pufferfish and TTX-bearing organisms, and the accumulation of relevant data is expected in the future.

## 4. Materials and Methods

### 4.1. Pufferfish Specimens

As shown in Table 3, individuals of the pufferfish *Takifugu alboplumbeus* were collected using hand nets from the spawning grounds of the pufferfish at two coastal sites (Figure 1) in Enoshima, Kanagawa, Japan (BW: 23.0–86.4 g for females, 21.1–68.7 g for males) and in Kamogawa, Chiba, Japan (BW: 38.0–86.7 g for females, 19.9–53.9 g for males) in June 2022. The collected pufferfish were determined to be male if sperm were released by gently squeezing their abdomen, and female if eggs were released. All individuals for TTXs analysis were euthanized by transection of the spinal cord after anesthesia with ice water, dissected, and skin, liver, gonad and the remaining tissues (‘others’), and were stored at −30 °C until TTX extraction. The remaining individuals not used for TTXs analysis were released at the collection site after confirmation of sex.

GSI of each fish was calculated from its gonad weight (GW) and BW using the following equation:

GSI = (GW/BW) × 100

### 4.2. LC-MS/MS Analysis

The tissue from one individual was homogenized, and approximately 0.2 g of the homogenate was used for extraction of TTXs according to the method of Suo et al. [11]. The extract was filtered through a 0.45 μm Supra Pure Syringe Filter (Recenttec, Taipei, Taiwan), and LC-MS/MS analysis was performed on a LC-20AD solvent delivery system (Shimadzu, Kyoto, Japan) connected to a X500R Q-TOF Mass Spectrometer (SCIEX, Framingham, MA, USA) with an ESI ion source. Following the method of Suo et al. [11], the sample solutions were analyzed by multiple reaction monitoring (MRM) mode at a flow rate of 0.3 mL/min on an Atlantis HILIC Silica column (Waters, Milford, MA, USA). The optimized transitions for qualification and quantification were *m*/*z* 320.1 > 302.1 and *m*/*z* 320.1 > 162.06 for TTX, respectively, while they were *m*/*z* 272.1 > 254.1 and *m*/*z* 272.1 > 162.1 for 5,6,11-trideoxyTTX, respectively. The calibration curve was created using 1–50 ng/mL of standard for TTX (FUJIFILM Wako Pure Chemical, Osaka, Japan) and 5,6,11-trideoxyTTX [39], and showed good linearity and precision (TTX: *y* = 445.36012*x*, *r*^2^ = 0.99; 5,6,11-trideoxyTTX: *y* = 168.20873*x*, *r*^2^ = 0.99).

### 4.3. Statistical Analysis

The statistical significance of the difference in BW was analyzed by one-way analysis of variance followed by Tukey Honestly Significant Difference test, while differences in toxin levels were analyzed by the Kruskal–Wallis test followed by the Steel–Dwass test. Significance levels of 0.05 were set. Data are given as mean ± standard deviation.

## Figures and Tables

**Table 1:** Localization of TTX and 5,6,11-trideoxyTTX in male and female *Takifugu alboplumbeus*.

Locality	Sex	No. of Specimen	TTX ^1^					5,6,11-trideoxyTTX ^1^				
			Total Amount (μg)	Tissue Distribution (%)				Total Amount (μg)	Tissue Distribution (%)			
				Skin	Liver	Gonad	Others		Skin	Liver	Gonad	Others
Kamogawa	Male	11	573 ± 294 ^*a*^	36.8 ± 21.2	31.2 ± 13.8 ^*a*^	5.7 ± 4.2 ^*b*^	26.4 ± 12.8 ^*a*^	337 ± 197 ^*a*^	69.9 ± 18.8 ^*a*^	9.5 ± 7.7 ^*a*,*b*^	2.4 ± 1.9 ^*a,b*^	18.2 ± 11.6 ^*a*,*b*^
	Female	9	734 ± 799 ^*a*^	25.0 ± 11.1	12.6 ± 6.4 ^*b*^	54.3 ± 15.0 ^*a*^	8.0 ± 6.1 ^*a,b*^	667 ± 846 ^*a*^	47.2 ± 18.2 ^*a*,*b*^	1.6 ± 0.7 ^*b*,*c*^	46.7 ± 20.8 ^*a*^	4.5 ± 5.7 ^*b*^
Enoshima	Male	14	301 ± 252 ^*b*^	44.4 ± 18.4	33.7 ± 12.6 ^*a*^	1.6 ± 0.7 ^*c*^	20.2 ± 8.4 ^*a*^	117 ± 69 ^*b*^	71.4 ± 17.0 ^*a*,*b*^	6.2 ± 10.7 ^*a*^	1.7 ± 1.2 ^*b,c*^	20.8 ± 7.9 ^*a*^
	Female	12	267 ± 301 ^*b*^	30.8 ± 13.5	2.0 ± 4.0 ^*c*^	59.2 ± 18.3 ^*a*^	7.9 ± 10.5 ^*b*^	211 ± 216 ^*a*,*b*^	38.8 ± 20.4 ^*b*^	0.5 ± 0.6 ^*c*^	54.7 ± 23.8 ^*a*^	6.1 ± 5.7 ^*b*^

^1^ Data are presented as means ± standard deviation. Different alphabetical letters (*a*–*c*) indicate significant differences among the measured values in the column (*a* > *b* > *c*, Steel–Dwass test, *p* < 0.05).

**Table 2:** TTX/5,6,11-trideoxyTTX ratio in tissues from *Takifugu alboplumbeus* ^1^.

Locality	Sex	Skin	Liver	Gonad	Others	Whole Body
Kamogawa	Male	1.1 ± 0.7	24.2 ± 25.9	3.8 ± 2.1	3.5 ± 3.3	2.0 ± 0.8
	Female	0.9 ± 0.4	9.0 ± 6.8	1.8 ± 0.8	1.6 ± 0.8	1.4 ± 0.5
Enoshima	Male	1.7 ± 0.9	15.3 ± 11.4	2.2 ± 1.5	2.6 ± 1.5	2.8 ± 1.6 *^a^*
	Female	1.3 ± 0.9	4.4 ± 2.3	1.9 ± 1.4	1.5 ± 0.7	1.3 ± 0.4 *^b^*

^1^ Data are presented as means ± standard deviation. Different letters on values significant differences between the measured values (*a* > *b*, Steel–Dwass test, *p* < 0.05).

**Table 3:** *Takifugu alboplumbeus* specimens used in this study ^1^.

Locality	Sex	No. of Specimen	Body Weight (g)	Tissue Weight (g)			
				Skin	Liver	Gonad	Others
Kamogawa	Male	11	36 ± 11 *^b^*	4.4 ± 1.0	1.3 ± 0.5	6.1 ± 2.5	25 ± 7.2
	Female	9	56 ± 14 *^a^*	7.4 ± 1.8	2.0 ± 0.7	6.4 ± 4.1	40 ± 10
Enoshima	Male	14	38 ± 12 *^b^*	4.7 ± 1.3	1.8 ± 1.3	6.0 ± 2.3	26 ± 7.6
	Female	12	56 ± 16 *^a^*	7.0 ± 2.1	2.8 ± 1.5	7.5 ± 5.3	39 ± 11

^1^ Data are presented as means ± standard deviation. Different letters on values significant differences between the measured values (*a* > *b*, Tukey Honestly Significant Difference test, *p* < 0.05).

## Data Availability

All data generated or analyzed during this study are included in this article and Supplementary Information.

## Supplementary Materials

The following supporting information can be downloaded at: https://www.mdpi.com/article/10.3390/md21040207/s1, Figure S1: Sex differences in the amount of TTX and 5,6,11-trideoxyTTX in skin, liver, gonad and other tissues (others) from the pufferfish *Takifugu alboplumbeus*; Preliminary study: TTX tolerance in moray eels *Gymnothorax kidako*; Table S1: Effect of different TTX concentration on survival of moray eel.
