# Supplementary material for: Levels of Tetrodotoxins in Spawning Pufferfish, Takifugu alboplumbeus
Source: Mar Drugs. 2023 Mar 25;21(4):207. doi: 10.3390/md21040207 (PMC10141859; doi:10.3390/md21040207)
Supplement: Supplementary file 1 [file marinedrugs-21-00207-s001.zip › Preliminary study.pdf]

Preliminary study. TTX tolerance in moray eels *Gymnothorax kidako*

To investigate TTX tolerance in moray eels *Gymnothorax kidako*, TTX was injected into the peritoneal cavity of the morays. Three individuals of *G. kidako* (660–1040 g body weight) were collected using an eel trap baited with fresh Pacific saury *Cololabis saira* in the coastal waters of Shimoda, Shizuoka, Japan (34°39'38"N, 138°58'56"E) in July 2014. These moray eels were kept for 24 hours in a flow-through seawater tank at the Shimoda Marine Experimental Station of Nihon University College of Bioresource Sciences. TTX was dissolved in 0.1% acetic acid to prepare an injection solution of 1 μg/μL. TTX solutions (258, 400, and 528 μL of 1 μg/μL) was injected into the peritoneal cavity of the moray eels weighing 860, 1040, and 660 g to achieve final concentrations of 300, 400, and 800 μg/kg body weight of moray eel, respectively. After TTX injection, moray eels were kept in the flow-through tank and observed. As shown in Table S1, the moray eels injected with up to 400 μg of TTX per kilogram of body weight survived even 24 hours after injection, whereas the individual injected with 800 μg of TTX per kilogram of body weight died at 25 minutes.

Table S1. Effect of different TTX concentration on survival of moray eel

Individual No.	Body weight (g) [1]	Injection volume (μL) [2]	Final concentration of TTX (μg/kg) [3]	Status
01	860	258	300	Survived
02	1040	400	400	Survived
03	660	528	800	Death after 25 minutes

[1] Body weight of moray eel used in this study.

[2] A TTX solution of 1 μg/μL was used.

[3] The concentration of TTX per kilogram of body weight in moray eels is presented.
