# Supplementary material for: Levels of Tetrodotoxins in Spawning Pufferfish, Takifugu alboplumbeus
Source: Mar Drugs. 2023 Mar 25;21(4):207. doi: 10.3390/md21040207 (PMC10141859; doi:10.3390/md21040207)
Supplement: Supplementary file 1 [file marinedrugs-21-00207-s001.zip › Suppl figS1_md.pdf]

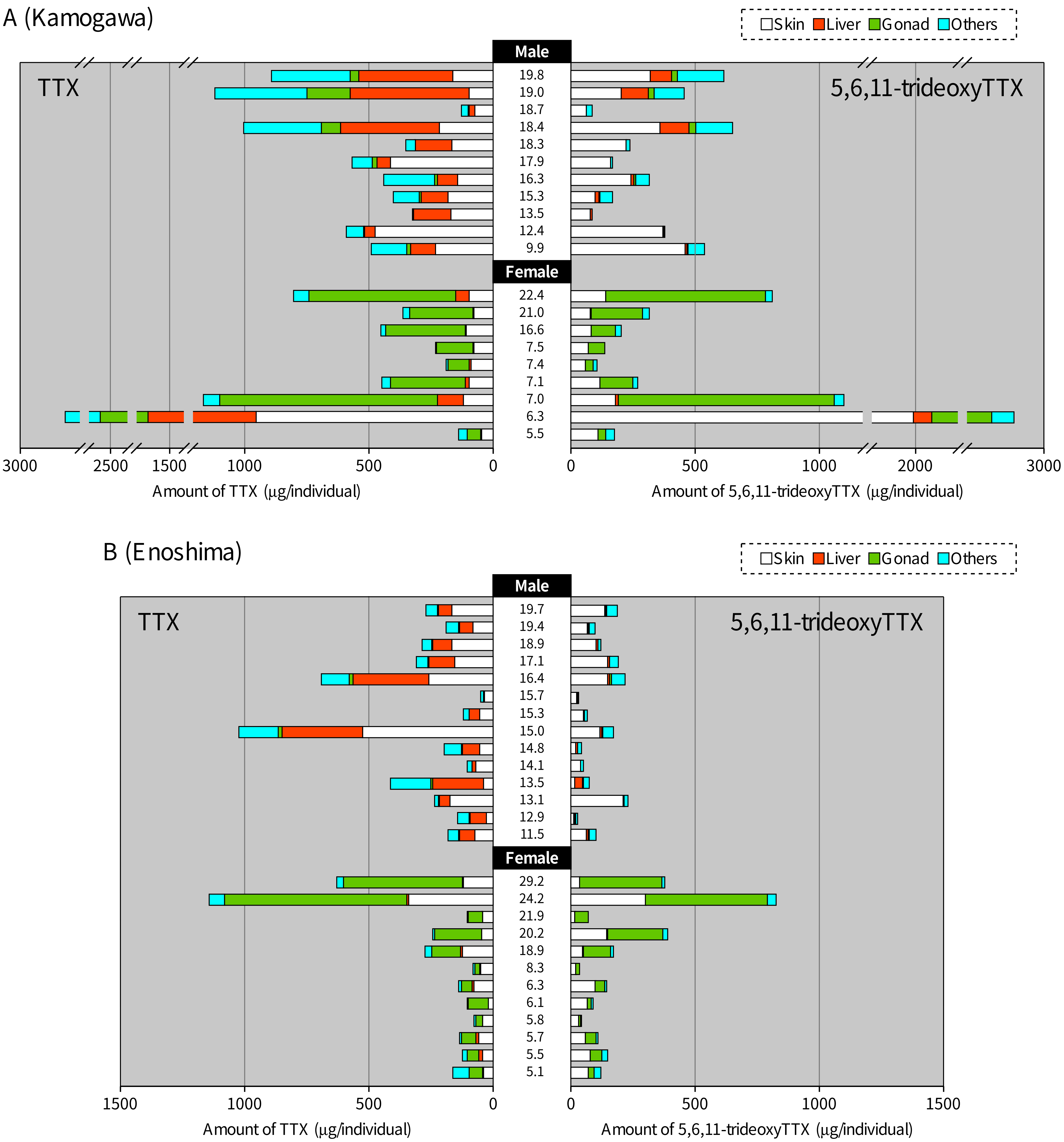

Figure S1. Sex differences in the amount of TTX and 5,6,11-trideoxyTTX in skin, liver, gonad and other tissues (others) from the pufferfish Takifugu alboplumbeus. Panels A and B represent data from individuals from Kamogawa and Enoshima, respectively. The numerals between the TTX (left side) and 5,6,11-trideoxyTTX bars (right side) indicate the GSI of the individual.
